# Investigation of Goubau Wave Propagation on Large Pipes for Sensing Applications

**DOI:** 10.3390/s23114991

**Published:** 2023-05-23

**Authors:** David W. Greve, Jagannath Devkota, Paul R. Ohodnicki, Ruishu Wright

**Affiliations:** 1 National Energy Technology Laboratory, Pittsburgh, PA 15236, USA; 2 Department of Electrical and Computer Engineering, Carnegie Mellon University, Pittsburgh, PA 15213, USA; 3 NETL Support Contractor, Pittsburgh, PA 15236, USA; 4 Department of Materials Science and Mechanical Engineering, University of Pittsburgh, Pittsburgh, PA 15260, USA

**Keywords:** Goubau, surface acoustic wave, sensing, launcher, wave propagation

## Abstract

We examine the application of guided waves on a single conductor (Goubau waves) for sensing. In particular, the use of such waves to remotely interrogate surface acoustic wave (SAW) sensors mounted on large-radius conductors (pipes) is considered. Experimental results using a small-radius (0.0032 m) conductor at 435 MHz are reported. The applicability of published theory to conductors of large radius is examined. Finite element simulations are then used to study the propagation and launching of Goubau waves on steel conductors up to 0.254 m in radius. Simulations show that waves can be launched and received, although energy loss into radiating waves is a problem with current launcher designs.

## 1. Introduction

Waves propagating on a single cylindrical conductor have been known since the publication by Sommerfeld [1,2]. Application of such conductors in transmission lines has been suggested by Goubau both with and without a dielectric region surrounding the conductor [3]. Apparently, the effect of the dielectric coating was examined much earlier by Harms [4]. The several names associated with early work has led to this wave mode being referred to by different names [5,6]; for simplicity, here we simply refer to Goubau waves.

A transmission line conveying such a wave has the potential advantage of low attenuation [3]. In contrast to other waveguides, the fields extend a significant distance from the conductor, resulting in both additional attenuation mechanisms and potential sensing applications. The unique physical extension of the propagating wave away from the conductor into the surrounding medium has been applied in a number of specific applications, for example, ice sensing [7], mid-range power transmission [8], and communication [9,10].

To date, work on Goubau waves has focused on the propagation and application of these waves on wires or other small-radius conductors (relative to the wavelength of the electromagnetic fields), and theory has not specifically examined the validity of approximations when the radius is comparable to or larger than the free-space wavelength. This work is part of an ongoing effort to investigate the use of guided waves on or in structures already existing in the built environment, with a particular application to infrastructure monitoring. We consider here Goubau waves on conductors with radii of the order of a free-space wavelength and, in addition, include the effect of low surface conductivity typical of steel pipes. In addition, we consider the problem of launching waves on large conductors. Our objective is to investigate the potential of Goubau waves in sensing applications for existing or newly installed steel pipelines. The interrogation of passive transponder-type sensors (such as surface acoustic wave sensors) is one potential application. Interrogation of such sensors using free-space electromagnetic wave propagation is severely limited in range due to the $1/r^{4}$ dependence of the reflected signal, where r is the separation between the interrogator and sensor. Another potential application, not considered here, involves sensing the material surrounding a conductor. These applications require the development of launchers suitable for larger-diameter conductors.

## 2. SAW Interrogation Using Goubau Waves in the Small Conductor Limit

**Figure 1:**
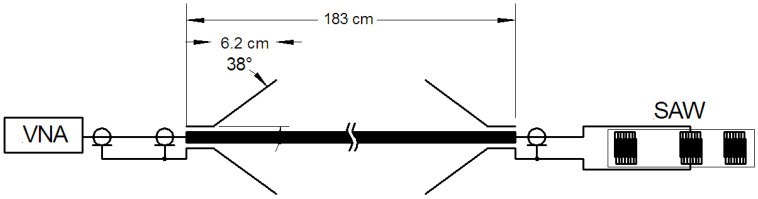
Schematic of arrangement for demonstration of the use of Goubau waves for interrogation. Some experiments used additional copper shields.

Figure 1 shows the arrangement for an experimental demonstration of the interrogation of SAW sensors using Goubau waves. A 0.0032 m radius brass conductor 1.83 m long has conical launchers at each end. One launcher is connected to a vector network analyzer (Keysight model P9373A), and the other is connected to a surface acoustic wave device described below. There is no other electrical connection to the SAW device, eliminating the possibility of ground loops.

**Figure 2:**
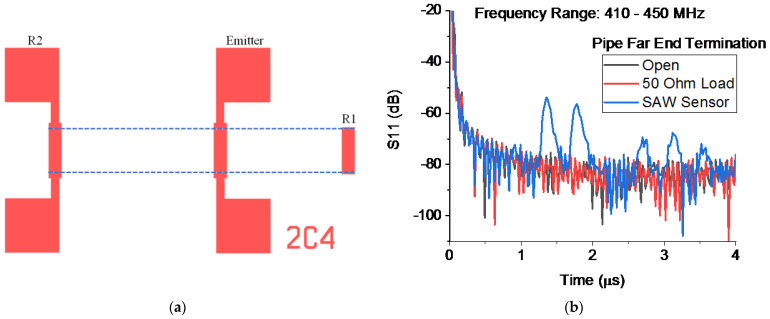
(**a**) SAW sensor mask layout and (**b**) S parameter S11 magnitude transformed into the time domain.

The SAW device (Figure 2a) consists of three interdigitated transducers (IDTs) on a Y-Z LiNbO_3_ substrate [11]. The period of the interdigitated transducers is 8 µm leading to an operating frequency of about 435 MHz. All IDTs have 30 finger pairs with an aperture of 780 μm. The IDT in the center is used as an emitter, and the two other IDTs spaced at 2.36 mm and 3.05 mm are open-circuited and operate as reflectors. When used as a sensor, the emitting IDT is excited by a windowed sinusoid with a center frequency of 435 MHz. Reflected pulses return to the emitter. In the figure, the propagation region for the acoustic wave is confined by dotted lines. The structures beyond the dotted lines do not affect the wave propagation. The delay time changes according to strain, temperature, etc., in the SAW device and can be detected and used for sensing. Here we replace the direct connection to the emitter with an RF link in order to demonstrate wireless interrogation using Goubau waves.

We observe the SAW reflections by using the vector network analyzer to measure the scattering parameter S11 as a function of frequency. Transforming S11(ω) into the time domain provides an equivalent way of observing the reflected pulses.

Figure 2b shows the transformed S11 using the experimental arrangement of Figure 1. We see the reflections from the two reflector IDTs (1.35 and 1.77 μs) along with multiple-bounce reflections (2.71, 3.12, and 3.54 μs). As expected, the reflections disappear when the SAW sensor is replaced with a resistive load.

Following the successful demonstration of small-conductor Goubau wave wireless sensor interrogation, we seek to apply a similar approach where a larger-radius conductor is used to support the Goubau waves. In the following sections, we examine the applicability of existing theory to large-radius conductors, particularly when the radius becomes comparable to the free-space wavelength.

## 3. Theory and Large-Radius Conductors

**Figure 3:**
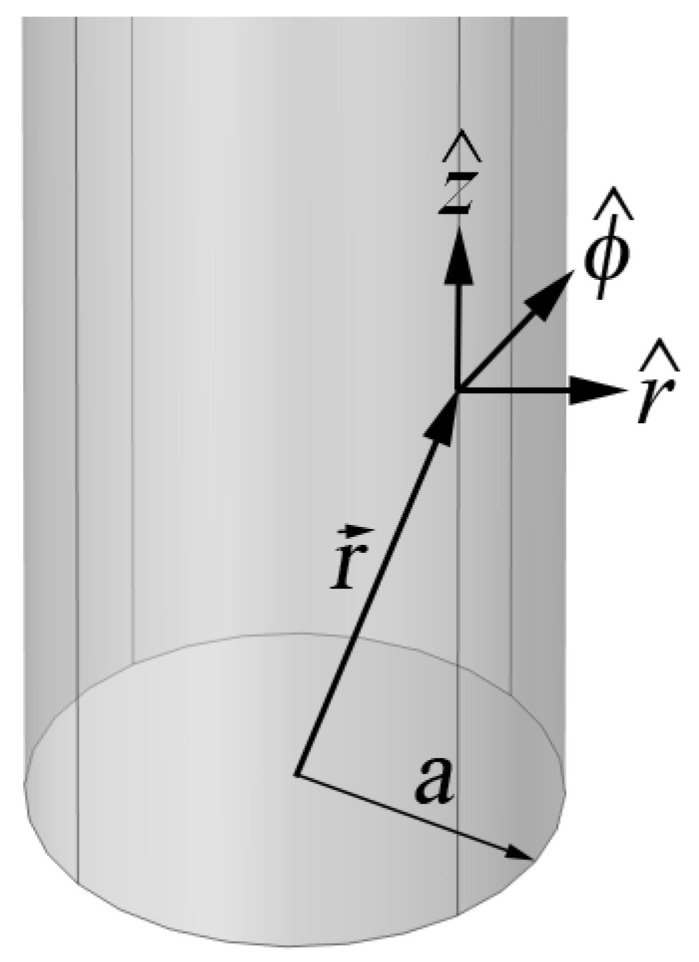
Coordinate system for conductor of radius *a* aligned with the *z* axis.

We summarize results from theory presented elsewhere [12] to examine its applicability for large pipes of lower conductivity. The Goubau mode field penetration is of the order of the conductor skin depth, so the wall thickness and contents of the pipe are irrelevant to the calculation We consider a cylindrical conductor with radius *a* in free space (Figure 3).

We consider a wave propagating along a cylindrical conductor in free space. For a wave traveling in the +z direction in cylindrical coordinates, the only nonzero component of the magnetic field is in the ϕ direction, thus

(1) $$H_{\phi }=H_{\phi }(r)e^{j(\omega t-hz)}$$

where h [1/m] is the wavenumber or propagation constant and *ω* [1/s] is the angular frequency. The solutions to Maxwell’s equations in cylindrical coordinates are Bessel functions. Inside the conductor, the solution decaying into the conductor is

(2) $$H_{\phi }=A\cdot J_{1}(\gamma_{c}r)$$

where $\gamma_{c}=\sqrt{\varepsilon_{c}\mu_{0}\omega^{2}-h^{2}}$ with $\varepsilon_{c}$ is the permittivity of the conductor and $\mu_{0}$ [H/m] is the permeability of free space. Outside the conductor, the appropriate solution that approaches zero at infinity is the Hankel function

(3) $$H_{\phi }=B\cdot H_{1}^{\left(1\right)}(\gamma_{a}r)$$

where $\gamma_{a}=\sqrt{\varepsilon_{0}\mu_{0}\omega^{2}-h^{2}}$ with $\varepsilon_{0}$ [F/m] the permittivity of free space. The values of h and $\gamma_{a,c}$ are determined by requiring continuity of $H_{\phi }$ and $E_{z}$ at the surface of the conductor. From $H_{\phi }$ and a Maxwell equation, we can find $E_{z}$ and $E_{r}$ as

(4) $$E_{r}=A\frac{h}{\omega \varepsilon_{c}}\cdot J_{1}(\gamma_{c}r)\;E_{r}=B\frac{h}{\omega \varepsilon_{0}}\cdot H_{1}^{\left(1\right)}(\gamma_{a}r)$$

(5) $$E_{z}=A\frac{\gamma_{c}}{j\omega \varepsilon_{c}}\cdot J_{0}(\gamma_{c}r)\;E_{z}=B\frac{\gamma_{a}}{j\omega \varepsilon_{0}}\cdot H_{0}^{\left(1\right)}(\gamma_{a}r)$$

where the first expressions apply inside the conductor and the second outside the conductor. One of the constants A and B is determined by the magnitude of the propagating field, and the other is determined when boundary conditions are imposed at the conductor surface. Imposing the continuity boundary conditions yields an equation that is best solved after making some approximations. Here we review these approximations and their validity, especially for moderate conductivity and conductors with large diameters.

### 3.1. Effective Permittivity Inside the Conductor

Inside the conductor $\nabla \times \vec{H}=j\omega \varepsilon_{0}\vec{E}+\sigma \vec{E}$, where σ [S/m] is the conductivity. For high conductivity and frequency not extremely high $\sigma \gg \omega \varepsilon_{0}$ and the conductor can be modeled with an effective complex permittivity $\varepsilon_{c}=-j\sigma /\omega$. This approximation is independent of conductor radius and is commonly applied when discussing the skin depth of conductors. At 1 GHz, $\omega \varepsilon_{0}$= 0.055 S/m, so this approximation is well satisfied even for poor conductors at VHF and microwave frequencies. Subject to this approximation, we have

(6) $$\gamma_{c}\approx (1+j)\sqrt{\sigma \mu_{0}\omega /2}=(1+j)/\delta$$

where δ is the skin depth.

### 3.2. Solution Inside the Conductor

The solution inside the conductor represents the decay of the magnetic field over a distance of the order of the skin depth. For macroscopic-size conductors, the skin depth is very small compared to the conductor radius. Consequently, the Bessel functions $J_{0}(\gamma_{c}r)$ and $J_{1}(\gamma_{c}r)$ can be approximated for large values of the argument as

(7) $$J_{n}(\gamma_{c}r)\approx \sqrt{\frac{2}{\pi \gamma_{c}r}}\cos\left(\gamma_{c}r-\frac{n\pi }{2}-\frac{\pi }{4}\right)$$

### 3.3. Applying the Boundary Conditions

Applying the continuity conditions for $H_{\phi }$ and $E_{z}$ at the surface of the conductor yields two equations. A nontrivial solution for A and B can be obtained only if

(8) $$\frac{J_{0}(\gamma_{c}a)}{J_{1}(\gamma_{c}a)}=\frac{\sqrt{\varepsilon_{0}\mu_{0}\omega^{2}-h^{2}}}{\sqrt{-j\sigma \omega \mu_{0}-h^{2}}}\cdot \frac{-j\sigma /\omega }{\varepsilon_{0}}\cdot \frac{H_{0}^{\left(1\right)}(\sqrt{\varepsilon_{0}\mu_{0}\omega^{2}-h^{2}}\cdot a)}{H_{1}^{\left(1\right)}(\sqrt{\varepsilon_{0}\mu_{0}\omega^{2}-h^{2}}\cdot a)}$$

For large values of $\gamma_{c}$, the ratio $J_{0}/J_{1}$ can be simplified using (7) as

(9) $$\frac{J_{0}(\gamma_{c}a)}{J_{1}(\gamma_{c}a)}=-j$$

This approximation only requires that the skin depth be small compared to the conductor radius, so it is valid for all cases of interest. For example, at frequency f=ω/2π=450 MHz and with a conductivity of 6× 10^5^ S/m we have δ = 3.06 × 10^−5^ m. The equation to be solved for h then becomes

(10) $$-j=\frac{\sqrt{\varepsilon_{0}\mu_{0}\omega^{2}-h^{2}}}{\sqrt{-j\sigma \omega \mu_{0}-h^{2}}}\cdot \frac{-j\sigma /\omega }{\varepsilon_{0}}\cdot \frac{H_{0}^{\left(1\right)}(\sqrt{\varepsilon_{0}\mu_{0}\omega^{2}-h^{2}}\cdot a)}{H_{1}^{\left(1\right)}(\sqrt{\varepsilon_{0}\mu_{0}\omega^{2}-h^{2}}\cdot a)}$$

**Figure 4:**
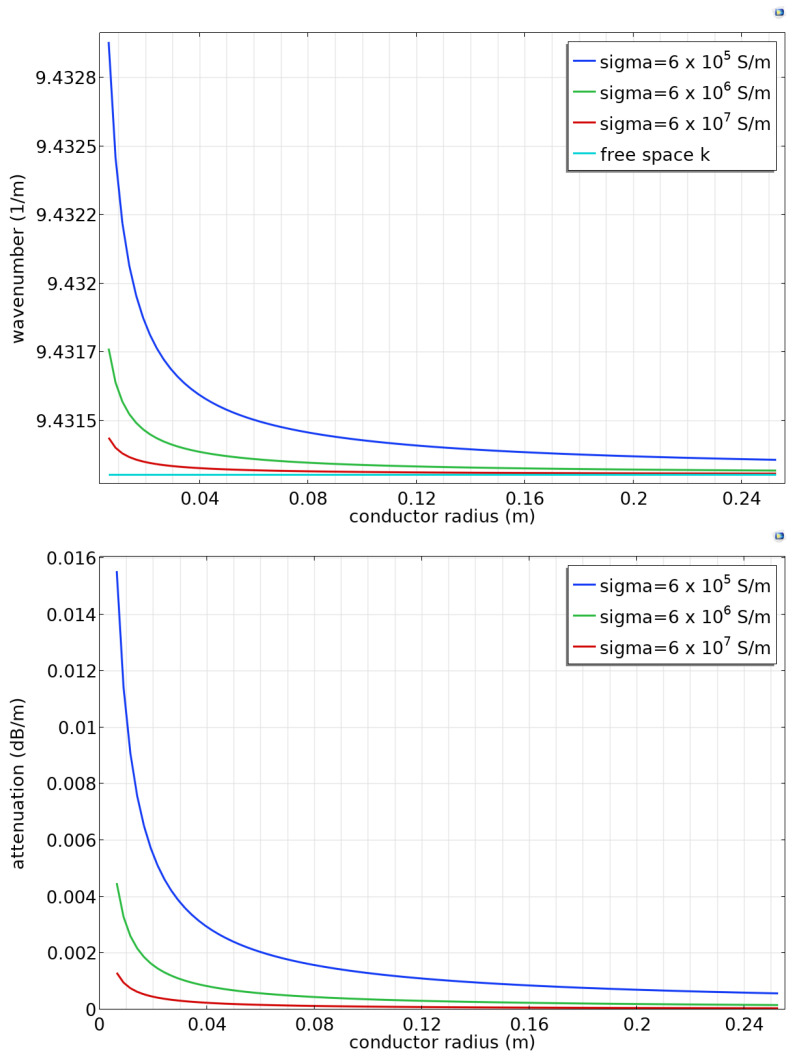
Analytic results for a steel conductor at 450 MHz (**top**) wavenumber or propagation constant and (**bottom**) attenuation in dB/m.

This equation can be solved numerically for the propagation constant h. The propagation constant is complex with a small imaginary part. Figure 4 (top) shows the real part of the calculated propagation constant at 450 MHz as a function of conductor radius. The propagation constant is nearly equal but slightly greater than the free-space wavevector, indicating that the propagation velocity is very slightly smaller than the free-space velocity of light. The wave velocity deviates more from the free-space velocity when the conductor radius is small or the conductivity is low. The imaginary part of the wavevector is related to the attenuation of the propagating wave (Figure 4, bottom). The attenuation is low, as expected [3], and decreases with increasing conductor radius.

We now consider the dependence of the fields $E_{r}$ and $H_{\phi }$ on radial position. For small values of the argument $\gamma_{a}r$, the Hankel function can be approximated as

(11) $$H_{1}^{\left(1\right)}(x)\approx \frac{2}{j\pi }\cdot \frac{1}{x}$$

This approximation results when only the leading term is considered in an expansion around z=0 [13] and is valid provided $\gamma_{a}r$ is small compared to unity. As noted above, h is very slightly greater than the free space wavenumber, and as a result, $\gamma_{a}$ is much smaller than the free space wavenumber. As a result, $\gamma_{a}r\ll 1$ within several wavelengths of the conductor, and this approximation is better when the conductor radius is large.

One important result from the analytic solution is the decay rate of the electric and magnetic fields. The magnetic field and the *z* component of the electric field decay according to 1/r. This relatively slow decrease means that propagation will be sensitive to objects and material properties near the conductor. This behavior is taken advantage of in some applications, particularly for sensing and diagnostics [7,9,10].

## 4. Finite Element Simulations

In the following sections, we use finite element simulation to answer questions that cannot be addressed using analytic approaches. We first demonstrate that simulations are consistent with the analytic results for wave velocity and field distributions. We then explore the launching and reception of Goubau waves.

The following simulations are for a conductor of steel with electrical conductivity 6 × 10^6^ S/m. The surrounding environment is air, and simulations are performed at 450 MHz. Simulations were performed using Comsol 6.0 using the electromagnetic waves (emw) module with an element size smaller than λ/4 (typically λ/10), where λ is the free-space wavelength. At the surface of the conductor, the impedance boundary condition [14,15] is applied, making it unnecessary to model the field penetration into the conductor.

### 4.1. Wave Velocity and Propagation

**Figure 5:**
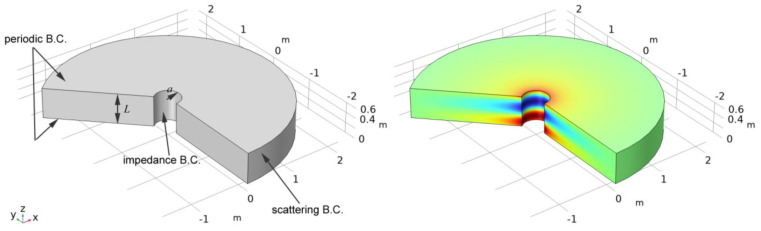
Eigenmode calculations for a domain with periodic boundary conditions: (**left**) geometry and (**right**) *r* component of the electric field for the lowest Goubau mode.

Consider a cylindrical domain surrounding a cylindrical conductor of radius a with length L (Figure 5, left). We impose periodic boundary conditions on the two caps. Eigenmode simulations yield the frequencies corresponding to wave modes with wavelengths L, L/2, etc. Using the frequency of the lowest Goubau mode, the phase velocity can be calculated as $v_{p}=L\cdot f$. (A similar approach is commonly used to determine wave velocities in surface acoustic wave devices [16,17]). Figure 5 (right) shows the radial component of the electric field for a conductor radius of 0.25 m.

A boundary condition must be imposed on the cylinder walls in order to approximate an infinite domain. This is particularly problematic with Goubau waves because the wave amplitude decays slowly with radial distance. There are well-known boundary conditions used to approximate an infinite domain for antenna and transmission line calculations; most commonly, the scattering boundary condition and a perfectly matched layer boundary condition [18]. Both are designed to eliminate reflections of waves incident on the boundary. However, neither is intended to allow the propagation of a wave parallel to the boundary without attenuation. Here we explore the consequences of using the scattering boundary condition on the wall.

**Figure 6:**
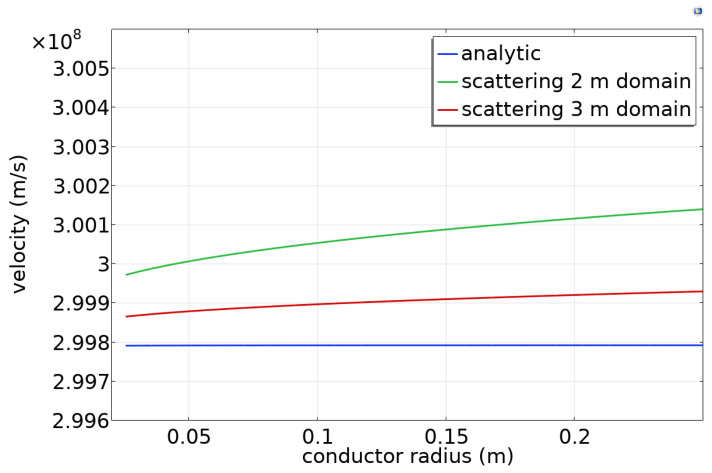
Wave velocity as a function of radius of a steel conductor.

Figure 6 shows the simulated phase velocity as a function of the conductor radius for a domain 0.6 m in length and 2 m larger in radius than the conductor radius. When the scattering boundary condition is used, the simulated phase velocity is slightly (0.1%) larger than the analytic result (Figure 6), and it becomes closer to the analytic result when the domain size is increased to 3 m. This indicates that the wall boundary condition has a small effect and becomes less important as the domain size increases.

We now consider the simulation of wave excitation in a long domain. A Goubau wave was excited at one end of a cylindrical domain using a user-defined port where the electric field in the r and z directions is specified using the analytic expressions of Section 3. The simulation is performed in cylindrical symmetry. The power received at the far end was calculated as a function of domain length. This simulation was performed for both 2 m and 3 m domain radii.

**Figure 7:**
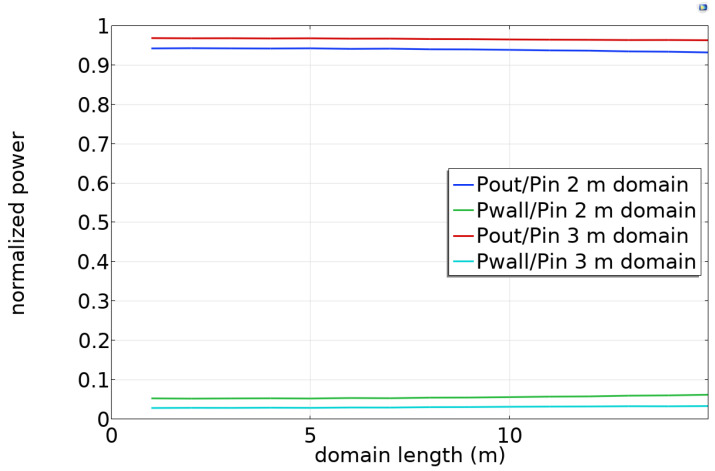
Power ratios for Goubau wave propagation in a finite-radius cylindrical domain.

Figure 7 shows the results for a 6 in (0.152 m) radius conductor with the conductivity of steel (6 × 10^6^ S/m). Almost all of the injected power is collected at the far end, with a small amount dissipated in the wall. Less power is lost when the domain radius is greater. Ideally, the attenuation would be far less than predicted by theory in Section 3, but the scattering boundary condition does not approximate an infinite domain well for Goubau waves. Nevertheless, the amount of attenuation is modest. We will use the scattering boundary condition in subsequent simulations, recognizing that the Goubau wave attenuation is considerably smaller in practice.

**Figure 8:**
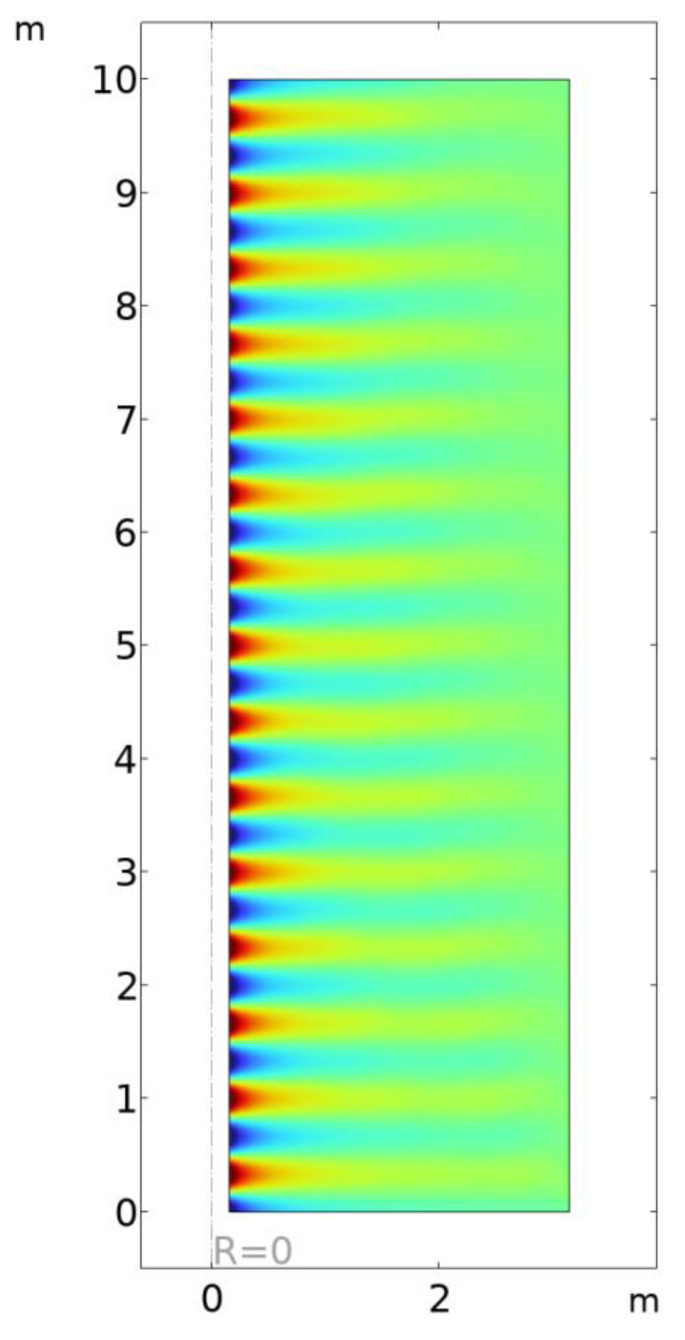
Plot on r-z plane of the r component of the electric field for Goubau wave propagation from bottom to top of a 10 m long cylindrical domain. The cylindrical conductor is steel with a radius of 0.152 m.

Figure 8 shows the radial electric field for a conductor 10 m in length surrounded by an air domain. The emitting port is at the bottom, and the transported power is calculated at the top port. The qualitative behavior is consistent with the analytic solution (Equations (4) and (5)).

**Figure 9:**
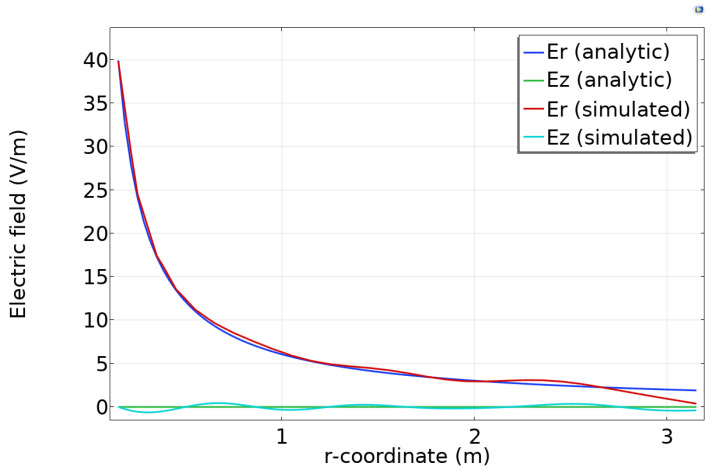
Analytic and simulated components of the electric field components at a constant z position.

Figure 9 compares the analytic and simulated electric fields for a particular z location along the domain. The simulated fields follow the analytic form except for minor ripples.

### 4.2. Conical Goubau Wave Launcher

Conical launchers have often been used to launch Goubau waves [3,19,20]. The conical launcher is a natural choice because of the similarity between the TEM wave mode in a coaxial feed and the TM fields in the Goubau mode.Here we explore the performance of conical launchers by simulation, including both large and small-radius conductors.

**Figure 10:**
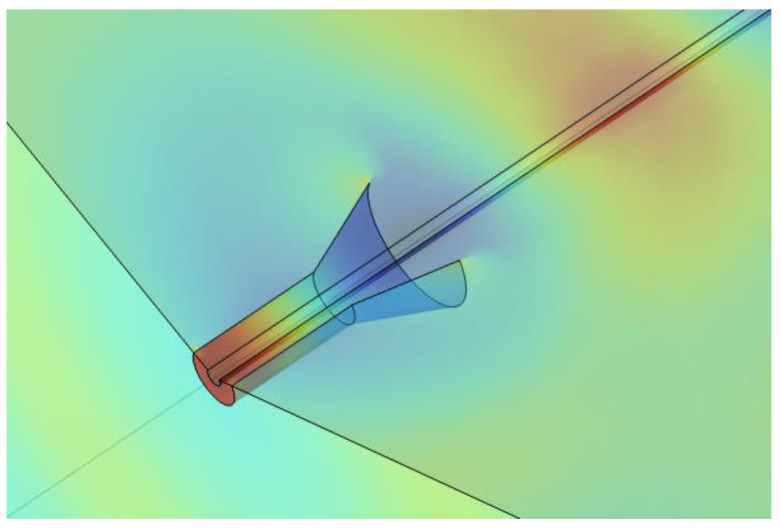
Geometry of cone launcher with coaxial feed.

We consider a coaxial line with a 50 ohm impedance surrounding the conductor that flares out to form a cone (Figure 10). Simulations have been performed for two cones spaced by about 10 m in a domain 3 m in radius. One cone is excited at 450 MHz (port 1), and the second is terminated at 50 ohms (port 2).

**Figure 11:**
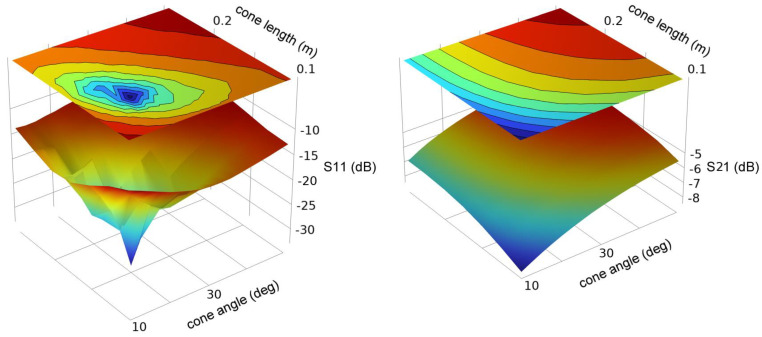
Scattering parameters S11 and S21 for a cone launcher on a 0.0127 m radius conductor as a function of cone angle and cone length.

**Figure 12:**
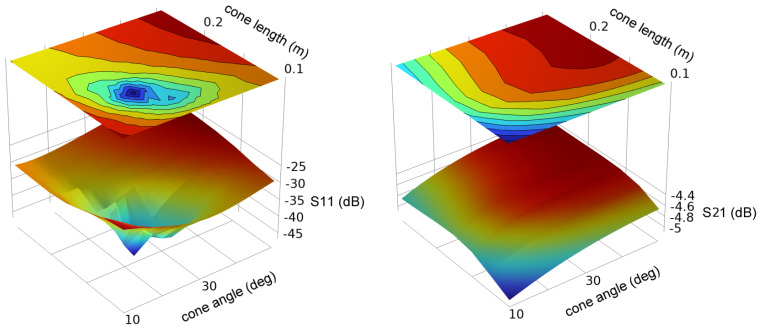
Scattering parameters S11 and S21 for a cone launcher on a 0.152 m radius conductor as a function of cone angle and cone length.

We first consider optimization of the cone geometry. Figure 11 and Figure 12 show the simulated S11 and S22 for conductor radii of 0.0127 m and 0.152 m.

Although there is a sharp optimum in the cone geometry, S11 remains relatively low (<−10 dB) for a wide range of dimensions. S21 is weakly dependent upon the cone dimensions and is near −6 dB (−5 to −8 dB) over the entire range of dimensions studied.

**Figure 13:**
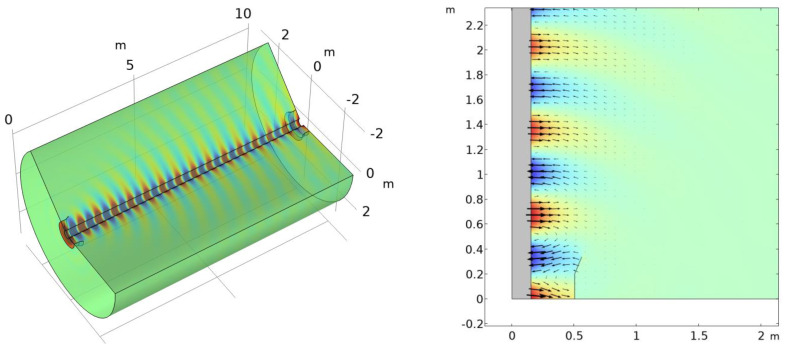
Cone launcher at 450 MHz and a steel conductor of radius 0.152 m: (**left**) *r* component of the electric field in a 10 m long domain terminated in a scattering boundary condition and (**right**) electric field vectors near the launcher.

The transmission loss, although small, is too large to be accounted for by loss at the walls. Figure 13 shows the radial electric field for a conductor radius of 0.152 m. In addition to the Goubau wave, some of the radiated energy is in the form of an approximately spherical wave that is radiated away from the cone. As this is the primary cause of attenuation, it is reasonable to use the scattering boundary condition for similar problems. The cone is an effective launcher, but the overall efficiency of coupling is limited by the modal mismatch causing the radiating component.

The cone launcher shown in this figure is impractical for large-radius conductors. It is difficult to arrange the feed so that the phase is equal on all parts of the cone. Moreover, the large insulator thickness required to match a 50 ohm impedance is unrealistic. In a subsequent section, we explore the potential of a partial-cone launcher.

### 4.3. Partial Cone Launcher

The coaxial-fed cone launcher discussed above is not appropriate for large-diameter conductors. The physical size of a cone surrounding the entire conductor is prohibitive, and in practice, it would be necessary to make a transition to an ordinary small-diameter coaxial cable. Accordingly, we have simulated a partial cone (one-quarter of a full cone) that is fed at a single point where a small-diameter coaxial cable can be attached.

**Figure 14:**
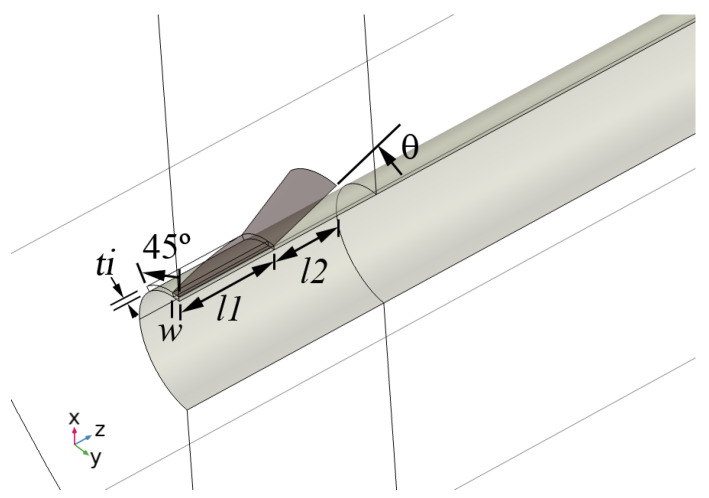
Geometry of partial-cone launcher.

Figure 14 shows the quarter-cone launcher, where one half of the symmetric geometry is shown. For simulation purposes, a symmetry boundary condition is imposed in the *x-z* plane. The launcher is driven by a lumped port of total width 2*w* that widens to form a taper of length *l*1. Between the taper and the large-diameter conductor is an insulator of thickness 0.012 m and relative permittivity ε*_r_* = 2. Parameters for several conductor diameters are shown in Table 1. The input impedance at the port is reactive, with a real part near 50 ohms (accounting for symmetry) and an inductive imaginary part. In the simulations reported below, a series capacitor is added to tune out the inductive part of the input impedance.

**Figure 15:**
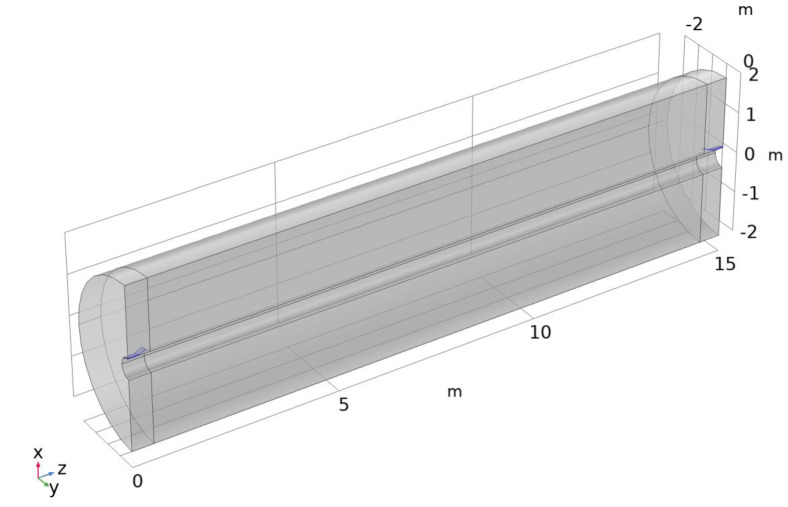
Geometry for transmission simulations.

Figure 15 shows the simulation domain, where the domain’s outer radius is 2 m. There are two partial-cone launchers, one driven at 450 MHz and the other terminated in a matched 50 ohm load. A scattering boundary condition is imposed on all outer surfaces. The cone and taper are modeled using the transition boundary condition (copper parameters), and the large-diameter circular conductor uses the impedance boundary condition (steel parameters, σ = 6 × 10^6^ S/m).

**Figure 16:**
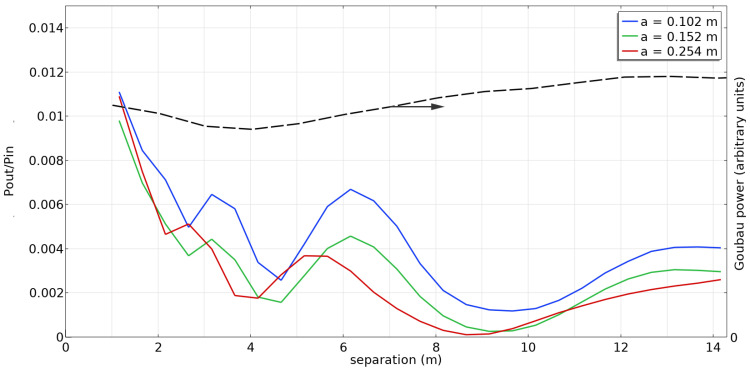
Power ratio as a function of separation between transmitting and receiving cone launchers (solid line) and estimate of the amount of power in the Goubau mode (black dashed line).

Figure 16 shows the simulated power transfer ratio P(out)/P(in) for three different conductor radii. The received power is considerably less than that obtained with a full-cone launcher. Nevertheless, it may be sufficient for some applications, particularly when compared with propagation through free space.

The peaks and valleys shown in Figure 16 are interesting features. This is believed to be due to interference between the Goubau wave propagated along the conductor and the spherical wave also emitted by the launcher.

We can illustrate the dependence of the amount of power contained in the Goubau wave on the propagation distance by an approximate mode analysis. We evaluate at the far surface the quantity

(12) $$\int_{endcap}\vec{E}\cdot {\vec{E}}_{Goubau}dS$$

where $\vec{E}$ is the electric field on the end cap obtained by simulation and ${\vec{E}}_{Goubau}$ is the field for the Goubau mode in the approximation of Equation (10). The result of this calculation is shown as the black dashed line in Figure 16 for a conductor radius of 0.102 m. The amount of power in the Goubau mode is roughly constant, consistent with the small attenuation seen in Figure 7.

It should be noted that the partial-cone launcher studied here is an obvious approach to consider but is also far from an optimal solution. Other designs should be explored and may very well lead to considerably improved power transmission.

## 5. Conclusions

The use of Goubau waves for wireless interrogation of SAW sensors has been demonstrated using a conductor with a small radius relative to the wavelength. We seek to extend this approach to conductors with a larger radius found in the built environment, such as pipes. The goal is to provide for sensor interrogation over longer distances than can be managed using free-space electromagnetic waves. This will require the development of launchers, which is best explored using simulation.

Finite element simulations of Goubau waves have been validated by comparison with analytic solutions. After confirming the applicability of approximations used in the theory for large-radius conductors relative to the wavelength, the mode shape and propagation velocity were compared with finite element simulations. In order to obtain good agreement, the appropriate boundary conditions must be used and the domain size must be sufficiently large.

The validated approach to simulation was then used to determine the effectiveness of full- and partial-cone launchers. Full-cone launchers are considered impractical for large-diameter conductors, and partial-cone launchers are inefficient, with a significant amount of energy radiated away from the conductor. The development of improved launchers would be desirable. We conclude that Goubau waves can be used to sense varying properties of the surrounding medium and also be used for telemetry and communication with distributed and passive wireless sensors.

## Figures and Tables

**Table 1:** Characteristics of partial-cone launcher.

Conductor Radius *a*	*l*1 (Line)	*l*2 (Taper)	ti (Insulator)	w (m)	2 ⋅ Zin
0.102 m	0.2 m	0.25 m	0.012 m	0.025 m	101 + j49 Ω
0.152 m	0.2 m	0.25 m	0.012 m	0.020 m	98 + j47 Ω
0.254 m	0.2 m	0.25 m	0.012 m	0.015 m	78 + j46 Ω

## Data Availability

Experimental data in Figure 2 is a screen capture and raw data is not available. Other figures present simulation results not experimental data.
